# Draft Genome Sequence of *Exiguobacterium* sp. KKBO11, Isolated Downstream of a Wastewater Treatment Plant in Houston, Texas

**DOI:** 10.1128/genomeA.00681-16

**Published:** 2016-07-21

**Authors:** Rupa Iyer, Ashish Damania

**Affiliations:** a Center for Life Sciences Technology, College of Technology, University of Houston, Houston, Texas, USA; b Department of Pediatrics-Tropical Medicine, Baylor College of Medicine, Houston, Texas, USA

## Abstract

*Exiguobacterium* sp. KKBO11, isolated near a wastewater treatment plant in Houston, Texas, USA, possesses a large number of genes involved in stress response and transport critical to survival in adverse environmental conditions. An unusually high copy number of RNA genes also possibly contributes to this microorganism’s versatility by promoting nutrient uptake.

## GENOME ANNOUNCEMENT

*Exiguobacterium* spp. are Gram-positive facultative anaerobic bacteria that are well known for extremophilic adaptations to temperature, salinity, pH, and other adverse conditions. *Exiguobacterium* spp. have been isolated from a number of exotic environments, including permafrost and hot springs, as well as common sources such as soil and plant rhizospheres (1). *Exiguobacterium* sp. KKBO11 was isolated from a brook downstream of a wastewater treatment plant and characterized as a putative organophosphate insecticide degrader through an environmental sampling research module conducted by University of Houston biotechnology undergraduates (2). An analysis of the KKBO11 sequencing project reveals that this strain is closely related to *Exiguobacterium* sp. MH3 and shares many of the metal resistance, transport, and nutrient uptake genes that are present in the MH3 strain (3). However, the KKBO11 strain has a slightly larger genome and possesses an unusually large complement of tRNAs in comparison to other sequenced *Exiguobacterium* spp. In addition, the copy number of rRNA genes present in KKBO11 is also atypical, with the majority of 16S and all of 23S rRNA sequences existing as partial copies interspersed throughout the genome. Taken together, the sequencing of this new strain is expected to not only help elucidate environmental stress response mechanisms in *Exiguobacterium* spp. but may also reveal an organism that could prove to be a viable candidate for agricultural bioremediation applications. The genome sequencing of KKBO11 was performed through Illumina MiSeq paired-end sequencing with a final sequencing coverage of 336.42x. Sequence reads were checked for quality using FastQC (4) and filtered using BBTools (5) with a minimum Phred score of 20. Paired-end reads were assembled into 60 contigs with the SPAdes version 3.7 program (6). Preliminary reference-based annotation using PATRIC (7) web resources was carried out to identify conserved pathways. Final *de novo* annotation was performed with Prokka (8) and the NCBI Prokaryotic Genome Automatic Annotation Pipeline (http://www.ncbi.nlm.nih.gov/genome/annotation_prok). The metabolic pathways of aromatic and heterocyclic compounds were examined through KEGG databases (9). This draft genome of strain KKBO11 comprises a total of 3,177,315 bp encoding for 3,204 protein-coding sequences, of which 44 are pseudogenes, 1,104 are hypothetical proteins, and 2,100 form known functional proteins. The genome has a GC content of 47.26% and contains an unusually high number of RNA loci, including nine, 13, 16 (5S, 16S, 23S) rRNAs, 70 tRNAs, and four ncRNAs.

### Nucleotide sequence accession numbers.

The *Exiguobacterium* sp. KKBO11 whole-genome shotgun project has the project accession number LUCU00000000. This version of the project (01) has the accession number LUCU01000000 and consists of sequences LUCU01000001 to LUCU0100060.
